# A hybrid linear discriminant analysis and genetic algorithm to create a linear model of aging when performing motor tasks through inertial sensors positioned on the hand and forearm

**DOI:** 10.1186/s12938-023-01161-4

**Published:** 2023-10-16

**Authors:** Veronica de Lima Gonçalves, Caio Tonus Ribeiro, Guilherme Lopes Cavalheiro, Maria José Ferreira Zaruz, Daniel Hilário da Silva, Selma Terezinha Milagre, Adriano de Oliveira Andrade, Adriano Alves Pereira

**Affiliations:** Postgraduate Program in Electrical and Biomedical Engineering, Faculty of Electrical Engineering, Centre for Innovation and Technology Assessment in Health, Federal University of Uberlândia, Uberlândia, Brazil

**Keywords:** Aging, Inertial sensors, LDA

## Abstract

**Background:**

During the aging process, cognitive functions and performance of the muscular and neural system show signs of decline, thus making the elderly more susceptible to disease and death. These alterations, which occur with advanced age, affect functional performance in both the lower and upper members, and consequently human motor functions. Objective measurements are important tools to help understand and characterize the dysfunctions and limitations that occur due to neuromuscular changes related to advancing age. Therefore, the objective of this study is to attest to the difference between groups of young and old individuals through manual movements and whether the combination of features can produce a linear correlation concerning the different age groups.

**Methods:**

This study counted on 99 participants, these were divided into 8 groups, which were grouped by age. The data collection was performed using inertial sensors (positioned on the back of the hand and on the back of the forearm). Firstly, the participants were divided into groups of young and elderly to verify if the groups could be distinguished through the features alone. Following this, the features were combined using the linear discriminant analysis (LDA), which gave rise to a singular feature called the LDA-value that aided in verifying the correlation between the different age ranges and the LDA-value.

**Results:**

The results demonstrated that 125 features are able to distinguish the difference between the groups of young and elderly individuals. The use of the LDA-value allows for the obtaining of a linear model of the changes that occur with aging in the performance of tasks in line with advancing age, the correlation obtained, using Pearson’s coefficient, was 0.86.

**Conclusion:**

When we compare only the young and elderly groups, the results indicate that there is a difference in the way tasks are performed between young and elderly individuals. When the 8 groups were analyzed, the linear correlation obtained was strong, with the LDA-value being effective in obtaining a linear correlation of the eight groups, demonstrating that although the features alone do not demonstrate gradual changes as a function of age, their combination established these changes.

**Supplementary Information:**

The online version contains supplementary material available at 10.1186/s12938-023-01161-4.

## Background

According to the World Health Organization (WHO), elderly are all individuals over the age of 60 years, however, when it comes to the formulation of public policies, this limit may vary in accordance with the conditions set by each country [1]. The share of elderly individuals is increasing worldwide, this is due to better living conditions, mainly related to health and nutrition that we are currently exposed to, and consequently increasing longevity [2].

It is estimated that in 2050, the elderly will make up 22% of the world’s population [3]. During the aging process there occur various changes, as for example the decline in cognitive functions and the performance of the muscular and neural systems, these changes make the elderly individual more susceptible to disease and death [4]. Aging is a common stage in all living organisms, but the manifestations in advancing age vary from individual to individual [5].

In [6], human aging is defined as a dynamic and adaptive process responding to external and internal damage over the course of life. The same authors group the consequences of aging into four domains: changes in body composition; balance between availability of energy and its demand; signaling networks that maintain homeostasis; and neurodegeneration.

The impact of aging on fine and precise movement is not well understood. However, several studies indicate that the physiological and biological changes related to aging affect the functional performance of both the lower and upper limbs, and consequently motor coordination in human beings [7].

According to [8], the lower members are specialized in gross motor skills related to mobility, while the main role of the upper members is focused on the strategic positioning of the hands. Therefore, the decline in motor coordination of the lower members as in the upper members increases dependency in the elderly regarding daily activities. According to [9], the upper members represent the active part of the human motor system, with it also being the most affected by aging, including the decline in motor performance during the execution of fine motor skills, which is, in itself, due to the deficit in coordination of hand movements. Due to such motives, in [10], the majority of studies that investigate the decline of the human motor system uses manual tasks, thus allowing the hands to act as a marker to verify the limitation in performed daily activities over the period of aging [9].

In research by [11], the function of the hand decreased with age in both men and women, especially after the age of 65. Anatomical and physiological alterations are present in the aging of the hands. According to [12], the function of the hand can be impaired by the degenerative loss of skeletal muscle mass (sarcopenia), thus causing a decline in both strength and resistance [12]. Therefore, the monitoring of the hands during the performing of manual tasks, can be a valuable tool for the monitoring of aging.

In this regard, the use of sensors placed on the hands to obtain objective measurements is of significant interest. In order to assist in understanding and characterizing the dysfunctions and limitations that occur due to neuromuscular changes related to advancing age, studies have suggested tools for the use of objective measures. As such, various types of sensor have been used for characterizing motor skills, among such one finds inertial sensors (accelerometer, gyroscope and magnetometer), which have been widely employed due to their size, low cost and ease of use [13–15]. The low cost associated with inertial sensors allows for their ease of purchase and the size and ease of use allow inertial sensors to be mounted and positioned onto different parts of the human body [16].

Inertial sensors have been applied in various studies. The research conducted in [17] proposes an algorithm for estimating physiological tremor by means of signals extracted from two inertial sensors, an accelerometer and a gyroscope.

In [13], the authors evaluated the movement from the plate to the mouth of individuals with Parkinson’s disease during feeding, the objective of this research was to evaluate movements associated with daily life via the collecting of signals from an accelerometer and a gyroscope. In the study by [18], accelerometers and gyroscopes were used to analyze the supination/pronation and elbow flexion/extension tasks.

From inertial sensor signals, information can be extracted by means of parameters, which can be analyzed and processed. One method that has been widely used for analyzing and processing signals is Machine Learning (ML); the parameters extracted through the inertial sensors are used as the classifier input. ML is a powerful support method in the investigation and prediction of motor alterations based on information extracted from biomedical signals via inertial sensors [19].

However, in many studies, the number of data signals may possess many dimensions, as a large quantity of dimensions can be detrimental to the performance of the ML algorithm, since the excessive number of features does not imply in a better learning of the model, learning is determined by the features that best describe the phenomenon to be analyzed or learned by the algorithm [20–22]. Additionally, according to [23] using few features may not be enough, but an excessive number of features can overload the computation process. Thus, the selection of an adequate set of features can enhance the classification of the algorithm and avoid collinearity between the data [23]. Therefore, it would be necessary to optimize the data dimension to improve the performance of the ML algorithm. There exist two main approaches for reaching this objective, the first is dimensionality reduction, the second approach is to combine the attributes, while trying to maintain data variability [19]. As such, linear discriminant analysis (LDA) is a tool used in statistics and ML to find the linear combination of features and dimensionality reduction [24, 25].

Set within this scenario, this study seeks to investigate which parameters manage to demonstrate a significant difference between groups of the young and elderly individuals by means of manual movements that are composed of three different motor tasks, traditional features and inertial sensors positioned on the hand and forearm. Although being one of the points of focus in our study, various other studies have covered the question of separation between groups of young and elderly individuals, but few studies have investigated gradual changes that occur in groups of different age ranges. Therefore, another approach adopted will be the use of a technique based on LDA for verifying if there exists a combination of traditional features that produce a linear correlation between the LDA value and aging, based on the study by [26]. It is expected that this study will contribute as a tool for predicting alterations in manual functionality.

## Results

### Comparison between young and elderly

Table 1 demonstrates the *p*-value of the features that managed to differentiate between the groups of young and elderly. Those features that did not demonstrate a significant difference between the groups are not presented in Table 1.

**Table 1:** Tasks, sensors and features that demonstrate significant difference between the groups of young and elderly after the application of the Mann–Whitney test

Task 1						
Features	Sensors					
	G1, *p*-value	G2, *p*-value	A1, *p*-value	A2, *p*-value	M1, *p*-value	M2, *p*-value
MAV						
MAVFD	0.019	< 0.001	< 0.001	< 0.001		
MAVSD	0.038	0.010	0.002	0.001		
RMS						
Peak						
ZC	< 0.001	< 0.001	0.001	0.001		
FMean	< 0.001	< 0.001	0.003	0.005		
FPeak						
F50	0.002	< 0.001	0.029	0.012		
F80	0.001	< 0.001	0.002	0.004		
Power3.5–7.5						
ApEn	0.003	0.002	< 0.001	0.027		
FuzzyEn	< 0.001	< 0.001	< 0.001	0.004		
VAR						
RANGE						
INTQ		0.036				
SKEWNESS	0.007					
KURTOSIS	0.016					

Task 2						
Features	Sensors					
	G1, *p*-value	G2, *p*-value	A1, *p*-value	A2, *p*-value	M1, *p*-value	M2, *p*-value
MAV	< 0.001	0.003	< 0.001	< 0.001	0.002	0.007
MAVFD	< 0.001	< 0.001	< 0.001	< 0.001	0.004	0.010
MAVSD	< 0.001	< 0.001	< 0.001	< 0.001	0.004	0.013
RMS	< 0.001	0.001	< 0.001	< 0.001	0.002	0.005
Peak	< 0.001	< 0.001	< 0.001	< 0.001	< 0.001	< 0.001
ZC				0.035		
FMean					0.005	
FPeak					0.006	
F50					0.013	0.018
F80					0.021	
Power3.5–7.5	< 0.001	< 0.001	< 0.001	< 0.001	0.001	0.007
ApEn					0.040	
FuzzyEn					0.010	
VAR	< 0.001	0.001	< 0.001	< 0.001	< 0.001	0.004
RANGE	< 0.001	< 0.001	< 0.001	< 0.001	< 0.001	0.003
INTQ	0.003	0.029	< 0.001	0.001	0.005	0.014
SKEWNESS	0.001	0.009		0.04		0.017
KURTOSIS	0.002	0.022				

Task 3						
	Sensors					
	G1, *p*-value	G2, *p*-value	A1, *p*-value	A2, *p*-value	M1, *p*-value	M2, *p*-value
MAV						
MAVFD						
MAVSD						
RMS						
Peak	0.034	0.014		0.017		
ZC			0.010	0.009		
FMean			0.012			
FPeak						
F50					0.018	0.045
F80			0.009	0.009		
Power3.5–7.5			0.024	0.017		
ApEn			0.012	0.006		
FuzzyEn						
VAR						
RANGE		0.022				
INTQ						
SKEWNESS	0.031	0.027	0.023	< 0.001		0.002
KURTOSIS	0.037			0.007		

**Fig. 1:**
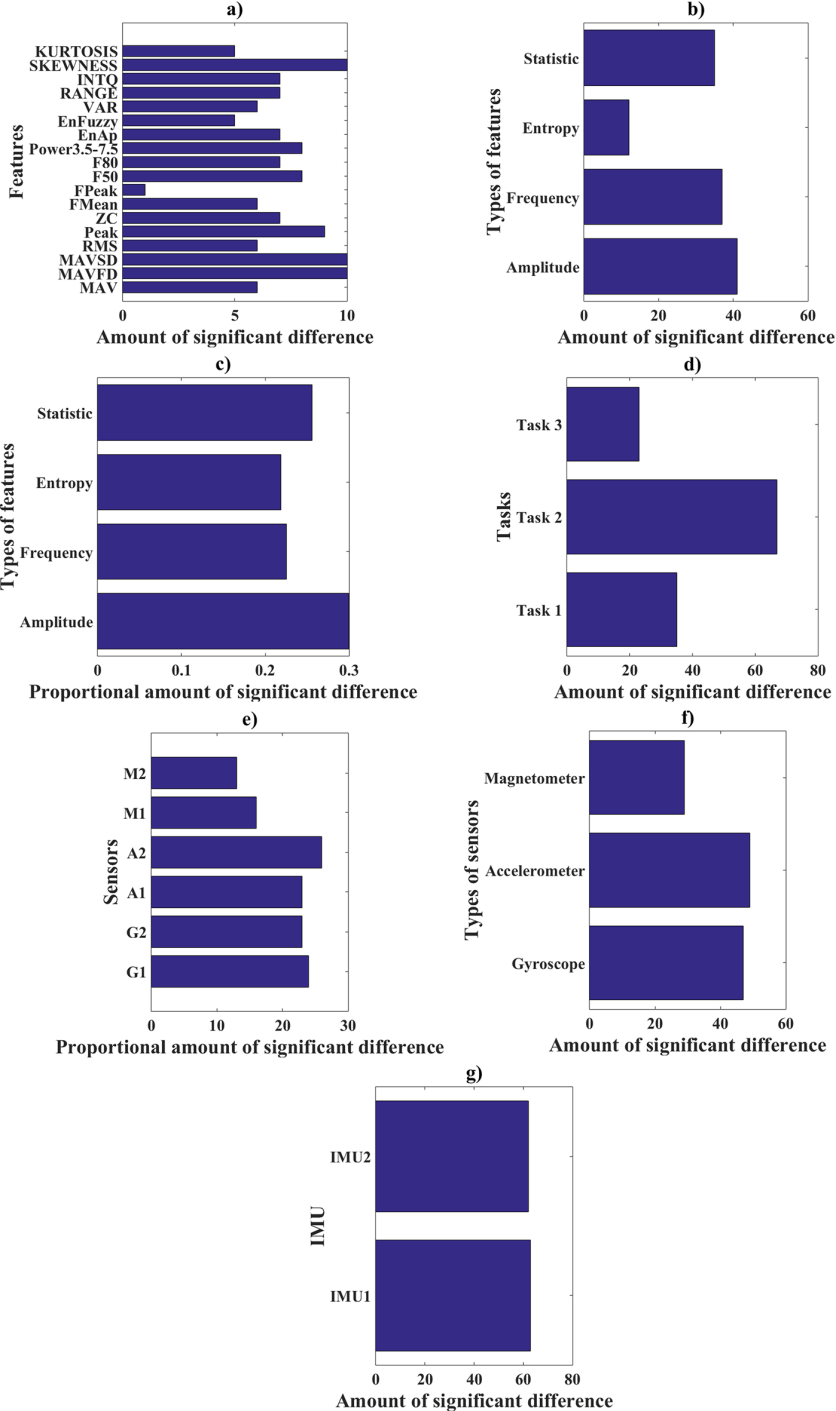
Quantity of significant differences per **a** features, **b** groups of features, **c** feature group proportion, **d** task, **e** sensors, **f** types of sensors, **g** IMU used

Through the analysis of Table 1, one notes that task 2 (pinch) presented the highest quantity of features that managed to differentiate the groups of young and elderly. Regarding the sensors, the magnetometer was that which presented the lowest capacity for differentiation between the groups of young and elderly. Figure 1 shows the quantity of significant differences presented by features, types of features, tasks, sensors and IMUs. Figure 1 shows the quantity of significant differences presented by features, types of features, tasks, sensors and IMUs.

Table 1 shows that 125 features are capable of individually differentiating between groups of young and elderly individuals. Despite various features managing to differentiate the groups of young from elderly, none managed to detect gradual changes over aging, while considering the 8 groups separately. As such, the features were combined to obtain a single feature called the LDA-value, based on the LDA [26]. The correlation between the LDA-value and age was investigated by means of the Pearson coefficient.

### LDA

The LDA opens the possibility for data classification and dimensional reduction, while projecting a multidimensional dataset onto a single dimension and after its application onto the dataset, a single value was obtained for each participant in each group. The algorithm, through the LDA and GA, considered 46 features relevant for the calculation of the LDA-value. The features highlighted with letters made up the group of relevant features for the calculation of the LDA-value, only the highlighted features were used for the calculation of the LDA-value. The letters were used to identify the contribution of each feature in Eqs. 23, 24 and 25. Table 2 demonstrates the features considered relevant for the LDA-value calculation.

**Table 2:** Relevant features for calculating the LDA-value

Task 1						
Features	Sensors					
	G1, *p*-value	G2, *p*-value	A1, *p*-value	A2, *p*-value	M1, *p*-value	M2, *p*-value
MAV	(a)	(f)				
MAVFD	(b)	(g)	(k)			(x)
MAVSD						
RMS						
Peak	(c)		(l)			(y)
ZC	(d)				(s)	
FMean	(e)	(h)	(m)			
FPeak		(i)		(o)	(t)	
F50				(p)		
F80				(q)		
Power3.5–7.5			(n)		(u)	(z)
EnAp				(r)	(v)	(A)
EnFuzzy						
VAR					(w)	
RANGE						
INTQ						
SKEWNESS		(j)				
KURTOSIS						

Task 2						
Features	Sensors					
	G1, *p*-value	G2, *p*-value	A1, *p*-value	A2, *p*-value	M1, *p*-value	M2, *p*-value
MAV						
MAVFD						
MAVSD						
RMS						
Peak						
ZC	(B)	(E)	(I)			
FMean		(F)				
FPeak	(C)			(K)		
F50		(G)	(J)	(L)		
F80						
Power3.5–7.5						
EnAp		(H)				
EnFuzzy						
VAR						
RANGE						
INTQ						
SKEWNESS	(D)			(M)		
KURTOSIS						

Task 3						
	Sensors					
	G1, *p*-value	G2, *p*-value	A1, *p*-value	A2, *p*-value	M1, *p*-value	M2, *p*-value
MAV						
MAVFD						
MAVSD						
RMS						
Peak						
ZC			(O)		(S)	
FMean						
FPeak		(N)				
F50						
F80						
Power3.5–7.5						
EnAp			(P)			(T)
EnFuzzy						
VAR						
RANGE						
INTQ						
SKEWNESS				(Q)		
KURTOSIS						(U)

**Fig. 2:**
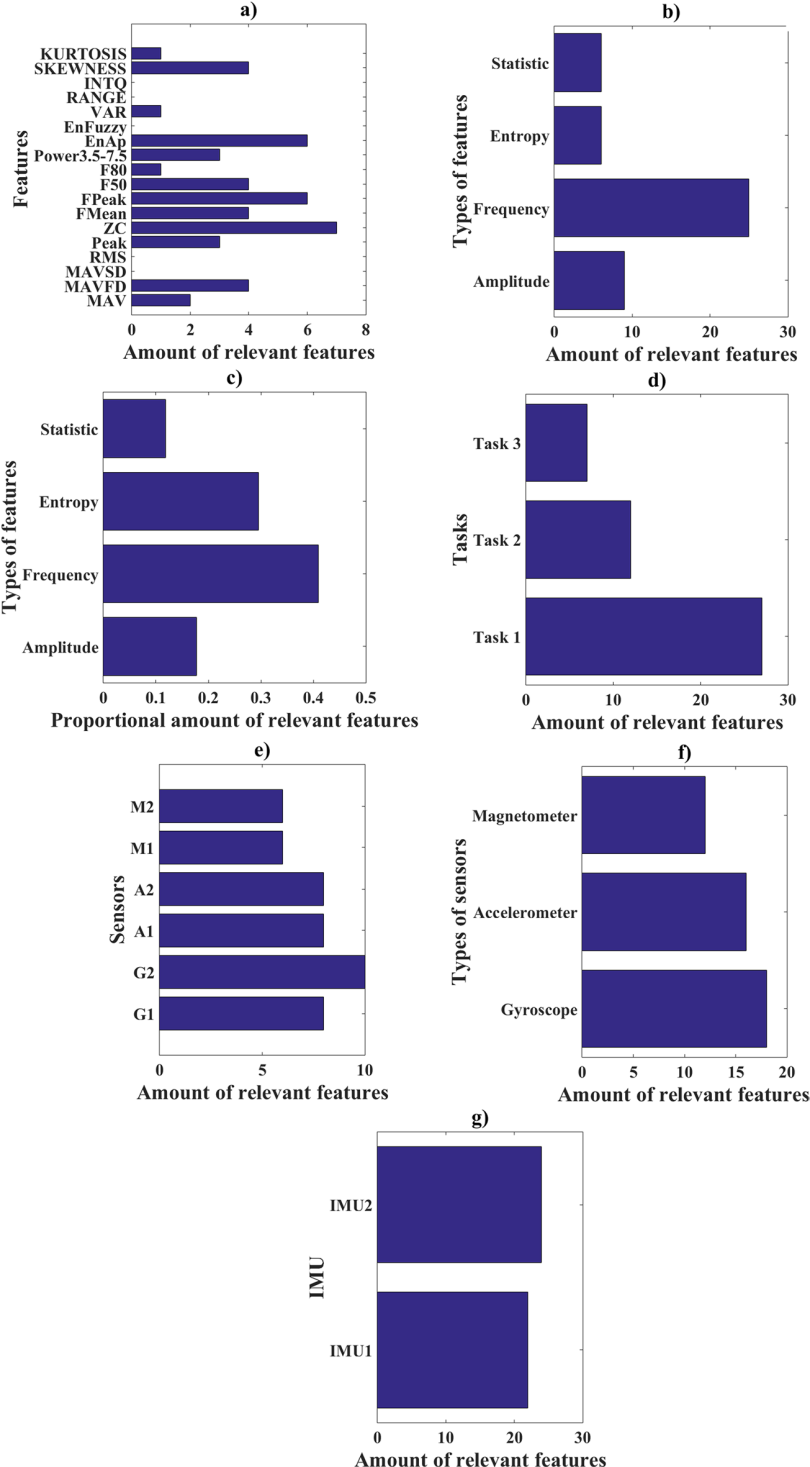
Quantity of relevant features by **a** features, **b** groups of features, **c** feature group proportion, **d** task, **e** sensors, **f** types of sensors, **g** IMU used

For the discrimination of young and old groups, only one feature (among the 125 highlighted in Table 1) is capable of performing the discrimination. However, for the creation of a linear aging model, 46 features were considered relevant. The relevant features, shown in Table 2, were inserted into Eqs. 23, 25 and 26. Figure 2 shows the 46 relevant features presented by feature, type of feature, tasks, sensors, and IMUs.

$$R=\left|\sqrt{{a}^{2}+{b}^{2}+{c}^{2}+\dots +{z}^{2}+{A}^{2}+\dots +{S}^{2}+{T}^{2}+{U}^{2}}\right|$$

$${\theta }_{1}={\mathrm{tan}}^{-1}\left(\frac{b}{a}\right); {\theta }_{2}={\mathrm{tan}}^{-1}\left(\frac{c}{\sqrt{{a}^{2}+{b}^{2}}}\right); {\theta }_{3}={\mathrm{tan}}^{-1}\left(\frac{d}{\sqrt{{a}^{2}+{b}^{2}+{c}^{2}}}\right);\dots ;$$

$${\theta }_{46}={\mathrm{tan}}^{-1}\left(\frac{U}{\sqrt{{a}^{2}+\dots +{T}^{2}}}\right)$$

$$LDA-value=R*\mathrm{cos}\left({\theta }_{1}+2.33\right)*\mathrm{cos}\left({\theta }_{2}+2.85\right)*\mathrm{cos}\left({\theta }_{3}+2.21\right)*\mathrm{cos}\left({\theta }_{4}+2.08\right)*\mathrm{cos}\left({\theta }_{5}+3.03\right)*\mathrm{cos}\left({\theta }_{6}+2.50\right)*\mathrm{cos}\left({\theta }_{7}+2.70\right)*\mathrm{cos}\left({\theta }_{8}+0.31\right)*\mathrm{cos}\left({\theta }_{9}+2.62\right)*\mathrm{cos}\left({\theta }_{10}+2.70\right)*\mathrm{cos}\left({\theta }_{11}+2.78\right)*\mathrm{cos}\left({\theta }_{12}+2.98\right)*\mathrm{cos}\left({\theta }_{13}+3.03\right)*\mathrm{cos}\left({\theta }_{14}+3.06\right)*\mathrm{cos}\left({\theta }_{15}+2.96\right)*\mathrm{cos}\left({\theta }_{16}+0.18\right)*\mathrm{cos}\left({\theta }_{17}+3.02\right)*\mathrm{cos}\left({\theta }_{18}+3.09\right)*\mathrm{cos}\left({\theta }_{19}+3.20\right)*\mathrm{cos}\left({\theta }_{20}+8.73\right)*\mathrm{cos}\left({\theta }_{21}-0.16\right)*\mathrm{cos}\left({\theta }_{22}-6.55\right)*\mathrm{cos}\left({\theta }_{23}+0.10\right)*\mathrm{cos}\left({\theta }_{24}+2.89\right)*\mathrm{cos}\left({\theta }_{25}+2.98\right)*\mathrm{cos}\left({\theta }_{26}+2.60\right)*\mathrm{cos}\left({\theta }_{27}+2.90\right)*\mathrm{cos}\left({\theta }_{28}+2.91\right)*\mathrm{cos}\left({\theta }_{29}+0.05\right)*\mathrm{cos}\left({\theta }_{30}+3.41\right)*\mathrm{cos}\left({\theta }_{31}+2.55\right)*\mathrm{cos}\left({\theta }_{32}+2.96\right)*\mathrm{cos}\left({\theta }_{33}+3.14\right)*\mathrm{cos}\left({\theta }_{34}+0.18\right)*\mathrm{cos}\left({\theta }_{35}-3.33\right)*\mathrm{cos}\left({\theta }_{36}-0.33\right)*\mathrm{cos}\left({\theta }_{37}+0.34\right)*\mathrm{cos}\left({\theta }_{38}+0.05\right)*\mathrm{cos}\left({\theta }_{39}+0.14\right)*\mathrm{cos}\left({\theta }_{40}+3.02\right)*\mathrm{cos}\left({\theta }_{41}-0.21\right)*\mathrm{cos}\left({\theta }_{42}+2.82\right)*\mathrm{cos}\left({\theta }_{43}+2.93\right)*\mathrm{cos}\left({\theta }_{44}+3.06\right)*\mathrm{cos}\left({\theta }_{45}+3.07\right)*\mathrm{cos}\left({\theta }_{46}+0.02\right)$$

The Kruskal–Wallis test using post hoc Bonferroni, was applied to the 8 groups regarding the LDA-value of each participant. Table 3 indicates the *p*-value among the groups, where the LDA-value has a significant difference.

**Table 3:** Results for the Kruskal–Wallis test in the comparison among the 8 groups

	Group 1	Group 2	Group 3	Group 4	Group 5	Group 6	Group 7	Group 8
Group 1	x	NS	0.021	< 0.001	< 0.001	< 0.001	< 0.001	< 0.001
Group 2	NS	x	NS	0.001	< 0.001	< 0.001	< 0.001	< 0.001
Group 3	0.021	NS	x	NS	0.004	< 0.001	< 0.001	< 0.001
Group 4	< 0.001	0.001	NS	x	NS	NS	< 0.001	< 0.001
Group 5	< 0.001	< 0.001	0.004	NS	x	NS	0.048	< 0.001
Group 6	< 0.001	< 0.001	< 0.001	NS	NS	x	NS	0.002
Group 7	< 0.001	< 0.001	< 0.001	< 0.001	0.048	NS	x	NS
Group 8	< 0.001	< 0.001	< 0.001	< 0.001	< 0.001	0.002	NS	x

*NS* No statistical difference

**Fig. 3:**
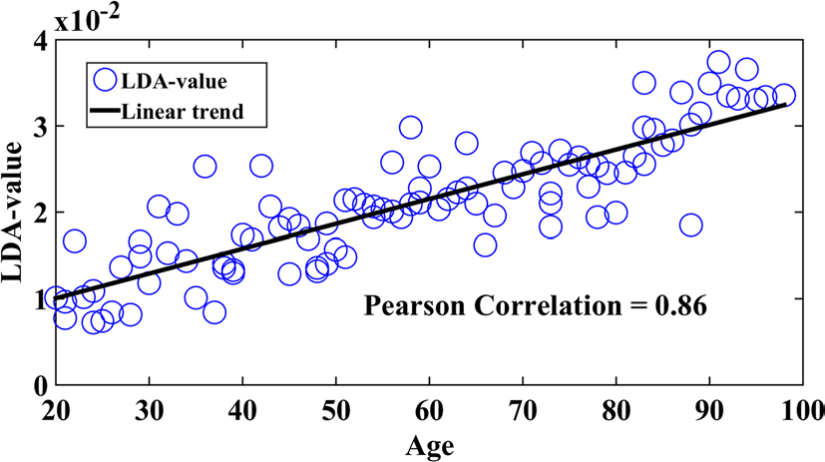
Graphic of the LDA-value vs age

Figure 3 presents the graphic for the age ranges regarding the LDA-value, where a relationship with a linear trend is observed.

## Discussion

The human body adopts a series of strategies to execute motor tasks, various studies cover the analysis of motor tasks with the aim of understanding and characterizing such strategies. However, many of such investigations are related to the analysis of individuals that suffer from neuromuscular disturbances, such as individuals with Parkinson’s disease. Studies of this nature, directed toward the elderly population, who also are subject to a decline in their capacity to perform motor tasks, are scarce. In addition, recent studies have demonstrated that the population of senior citizens is growing rapidly in a number of countries, thus giving this type of study importance in the maintenance of life quality for this portion of the population.

Motor tasks are performed in different ways, complexities, and parts of the body, but the human hand exercises and executes important functions in daily life, mainly in fine motor tasks. However, the biomechanical and neurophysiological execution of these motor tasks, for biological reasons related to aging, is compromised with age [27]. Therefore, the evaluation of tasks performed by the hands using inertial sensors, may aid in the understanding of changes that occur in the execution of motor tasks during the aging process.

### Sensor analysis

Various studies address the correlation between a signal collected from the individual and aging. These signals can arise from electromyography (EMG), electroencephalography (EEG), balance platforms, inertial sensors, cameras, and others [26, 28–30]. Among the various possibilities for signal acquisition, inertial sensors are becoming widely used for evaluating, classifying and characterizing aging, as these are cheap and can be applied to various executions of human movement [31, 32]. In the article from [33], an accelerometer and gyroscope were used to verify the decline of balance in the elderly, the authors verified changes in the parameters of inertial sensors, where the conclusion was reached that these changes may signify the decline in balance of elderly individuals.

Research by [34] used an accelerometer and a gyroscope to evaluate the correlation between parameters of human gait and individual traits. The authors found a strong nonlinear relationship between age and gait parameters.

The authors in [35] developed and evaluated a test for the detection of joint position based on a system composed of an accelerometer, a gyroscope and a magnetometer. In the study, one of the objectives was to evaluate the relationship between joint position proprioception and aging. The authors concluded that there is a decline in joint proprioception with age, this was reached by means of group comparison between the young and elderly.

In our study, we used two IMUs each composed of an accelerometer, a gyroscope, and a magnetometer. Through the analysis in Fig. 1, one verifies that the most frequent sensor in the discrimination of the groups between young and elderly was the accelerometer, localized on the back of the hand, conversely, the less frequent sensor was the magnetometer also located on the back of the hand. In regard to the type of sensor, the magnetometers were those which presented the lowest frequency of features in the discrimination between the young and elderly groups, but there was not any relevant difference found between the gyroscope and accelerometer. Among the IMUs, there was practically no difference between the number of features that discriminate the groups of young and elderly.

### Discrimination between young and elderly individuals

Initially, we evaluated the motor tasks to verify if the features used were able to discriminate the group of young participants from the elderly. This is a traditional analysis and has already been performed in a number of different ways. Our results corroborate with the results obtained in [36], in which evaluated cinematic features for analyzing the lie-to-stand (LTS) transfer, while describing differences related to age based on signals from inertial sensors positioned on the trunk, the authors compared a group of young individuals of ages between 20 and 50 years of age, and a group of elderly individuals with ages of over 60 years. As in our study, the results indicate that various features show significant differences between the groups, for instance, the duration of transference, acceleration, and maximum vertical velocity, but other features were unable to differentiate between the young and elderly groups. In our study, among the 324 features evaluated, 125 features were able to differentiate between the groups of young and elderly people.

In the study by [37], the objective was to define the main factors for good performance in anticipatory motor planning. The authors compared a group of young individuals, with ages between 19 and 28 years, and a group of elderly individuals aged between 61 and 86 years, the authors concluded that aging is associated with a sharp decline across all aspects of cognitive and motor functionality that were tested. The results of this study were similar to ours, as not all analyzed features were able to differentiate between young and elderly groups.

Most studies related to aging investigate only young and elderly groups. Our study differs from most approaches currently used in the literature and from studies by [36] and [37], as it seeks answers to questions related to changes in 8 different age groups.

### Parameters

In our study, we used 18 parameters to discriminate the individuals as young and elderly. We used parameters related to amplitude (RMS, Peak, MAV, MAVFD and MAVSD), 6 parameters related to frequency (ZC, FMean, FPeak, F50, F80 and Power3.5_7.5), 2 parameters related to signal entropy (ApEn and FuzzyEn) and 5 parameters related to signal statistics (VAR, Range, Intl, SKEWNESS and KURTOSIS), these parameters are well established and have already been used in part or in full in several other studies [38–42]. The parameters were combined to the 3 tasks and to the 6 sensors, thus resulting in a total of 324 features. A Pearson correlation greater than 0.9 of the 324 features was tested and 143 features were verified as presenting a correlation inferior to 0.9, among the remaining 143 features, 125 showed significant differences in the discrimination of the groups of young and elderly. Noteworthy here is that all the parameters of the signals used, across the task or sensor, showed a significant difference between the groups. By means of Fig. 1, one notes that three parameters are highlighted in the discrimination of the groups, with these being MAVFD, MAVSD and SKEWNESS, on the other hand, the Fpeak parameter was that which presented the lowest frequency among the parameters that managed to discriminate the groups of young and elderly individuals. In addition, proportionally and even in absolute values, the group of parameters with greater frequency in the discrimination of young and elderly groups was that of amplitude.

### Tasks

We used three tasks for evaluating aging regarding motor activity of the hand, we used the rest position [43, 44], the pinch task [45] and hand pronation/supination task [46]. By analyzing Fig. 1, one notes that the task of highest frequency in the discrimination between young and elderly groups was the pinch task.

To explain the results in biological terms is still a complex task, as the decline in motor activity during aging is an effect with an established knowledge, but its biological base is still little understood [47]. There exist various theories that try to explain aging, including genetic, non-genetic, autoimmune among other theories. However, none of the theories is absolute in explaining aging [48]. Then again, it is known that physiological, molecular and cellular changes occur [49] and that there is a strong relationship between the difficulty of performing the motor task with age [47]. Thus, the explanation concerning the pinch task having the highest frequency of features that can discriminate between young and elderly groups may be related to greater difficulty in performing this task.

### LDA-value

Despite various features having discriminated between the groups of young and elderly, no singular feature was capable of showing a correlation between aging across different age ranges and motor activity of the hand. The research developed by [26, 28, 29] also sought to correlate aging with signals from individuals, but the example from our study also did not manage a linear correlation with aging from any singular feature. As such, a combination of features was performed by means of the LDA-value.

Previous research has examined the linear relationship between ageing and performance on different tasks and features. The authors in [50] aimed to examine the alterations in running biomechanics that occur with advancing age. The study group consisted of participants ranging in age from 18 to 60 years. The findings of our research diverge from those reported in the study conducted by [50]. While the authors of that study were able to establish a linear association, the Pearson’s coefficient exhibited a relatively low value, peaking at 0.38.

In their study, Korhonen et al. [51] examined the decline in running performance associated with ageing. They analyzed biomechanical and skeletal muscle characteristics in a cohort of 77 male sprinters ranging in age from 17 to 82 years. Similar to our own study, the authors categorized the participants into different age groups. However, it is worth noting that the age thresholds used to form these groups differed from those employed in our investigation. The researchers conducted an analysis on five distinct groups that were categorized based on age ranges. These groups were designated as Group 1 (17 to 33 years), Group 2 (40 to 49 years), Group 3 (50 to 59 years), Group 4 (60 to 69 years), and Group 5 (70 to 82 years). The researchers obtained a Pearson correlation coefficient value of up to 0.77.

In our research, we observed a strong positive linear correlation (*r* > 0.86) between the LDA-value and the process of ageing. Our findings support the conclusions drawn by a previous study conducted by [26], where the authors examined the relationship between tremor and several balance-related aspects. The authors of that study reported a Pearson’s coefficient of 0.91 to quantify the relationship. The findings of a previous study [28] exhibit similarities to our own research. In that particular study, the association between ageing and features associated with the Archimedes spiral was examined. The Pearson coefficient reported in their work was 0.83, which aligns with our own results. In study [29], the authors aimed to establish a linear relationship between ageing and electroencephalography (EEG) features. They found that the Pearson’s coefficient, which measures the strength and direction of the association, was larger than 0.83, similar to our own findings. Similar to the findings of [26, 28, 29], our results indicate a decrease in the performance of motor tasks as individuals age, as proposed in this study. The utilization of the LDA-value, in conjunction with inertial sensors, is anticipated to provide a significant contribution in monitoring the progression of ageing, with the ultimate goal of ensuring enhanced quality of life and effective resource planning.

## Conclusion

In this study, we investigated motor tasks in two different ways. The first approach addressed the discrimination between young and elderly individuals, the results showed that there exists a significant difference in the value of features between the groups analyzed. The second approach verified if the combination of features (using the LDA-value) would produce a linear correlation between the LDA-value and the different age groups utilized. The linear relationship between the LDA-value and the different age groups, presented in Fig. 3, arrives at the consideration that the reduction in motor activity is directly associated with the age of the individual considered in this study and that this parameter could be employed for the characterization, follow-up and monitoring of a possible disorder that may affect the quality of life of such individuals. The LDA-value was shown to be efficient in presenting gradual alterations in the eight groups, thus demonstrating that despite isolated features not demonstrating alterations concerning age, the combination of these does evidence such alterations. Through the results, one arrives at the conclusion that the LDA-value is a relevant feature for the analysis of motor activity, with the potential for application in a variety of correlated studies in areas such as physiotherapy, geriatrics, and others.

## Methods

Data collected from 99 healthy individuals aged between 20 and 98 years, with no clinical evidence of neurological degeneration, were used. The dataset used in the experiments for this study were obtained by means of collection following the research protocol that was approved previously by the National Commission of Ethics in Research (CONEP) under CAAE 07075413.6.0000.5152. The study was conducted in accordance with the Declaration of Helsinki. All volunteer subjects signed a consent form before participating in the experiment.

The individuals were classified by separation into eight groups in accordance with their age range. The general features of the groups are described in Table 4, where *N* represents the number of individuals from each group.

**Table 4:** General features of the groups analyzed

Groups	Average age ± sd (years)	N
Group 1	24.53 ± 3.09	13
Group 2	35.17 ± 3.16	12
Group 3	45.15 ± 3.02	13
Group 4	54.87 ± 3.02	15
Group 5	64.45 ± 2.87	11
Group 6	74.71 ± 2.84	14
Group 7	84.53 ± 2.87	13
Group 8	93.62 ± 2.67	8

*sd* standard deviation

### Data collection

In order to perform the collection of data the TREMSEN (Precise Tremor Sensing Technology, INPI: BR 10 2014 023282 6) was employed, which was developed by researchers from the Center for Innovation and Technological Assessment in Health (NIATS)—(Núcleo de Inovação e Avaliação Tecnológica em Saúde (NIATS)), based at the Federal University of Uberlândia (UFU). The system is composed of two inertial measurement units (IMUs), the IMU was developed using the MinIMU 9 (ST Microelectronics, Switzerland) and the software utilized was developed in C# (Microsoft). The sensitivity of the gyroscope, accelerometer and magnetometer were set to ± 245°s, ± 2 g and ± 2 gauss, respectively, in accordance with studies by [16, 30, 52]. For Analogical/Digital conversion, a 12-bit converter of the microcontroller was employed as used in the TREMSEN (Atmel SAM3X8E ARM Cortex-M3). The signals were collected at a sampling frequency of 50 Hz. The data processing code was developed using Matlab and R-Studio.

The collections from participants were realized through two inertial units positioned on their upper dominant member, one of which (IMU2) was positioned onto the back of the hand aligned to the third finger, IMU2 refers to the signals from sensors Accelerometer 2 (A2), Gyroscope 2 (G2) and Magnetometer 2 (M2). The other IMU (IMU1) was positioned onto the distal third forearm, following the same alinement of IMU2, IMU1 refers to signals from sensors Accelerometer 1 (A1), Gyroscope 1 (G1) and Magnetometer 1 (M1), following from the area having been previously shaved and sanitized [30].

After the positioning of the sensors, the participants were instructed to perform the following tasks:

- Task 1—member static and the forearm in a semiflexion position.
- Task 2—with the forearm in the same position as (I), while performing the pulp-to-pulp pinch with all fingers.
- Task 3—supination and pronation of the forearm.

**Fig. 4:**
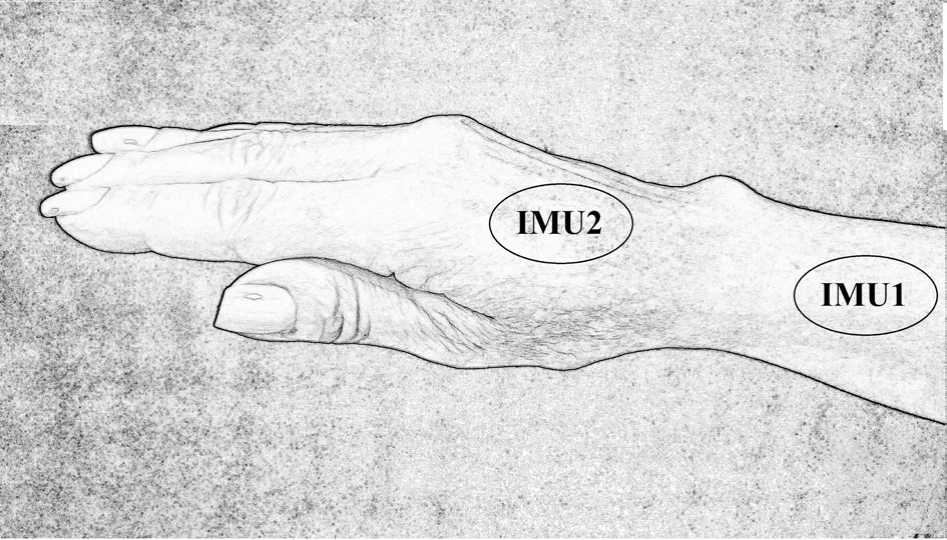
Positioning of the IMUs

All tasks had a minimum duration of five seconds. This protocol was performed three consecutive times for each participant. Figure 4 shows the localization of the IMUs.

**Fig. 5:**
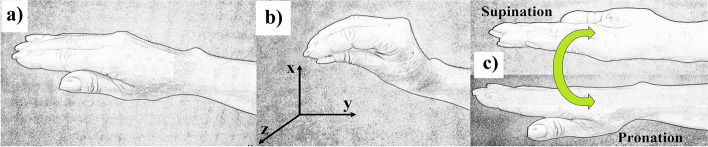
Executed tasks. **a** Task 1. **b** Task 2 and direction of axes—*x*: vertical; *y*: medial–lateral and *z*: anteroposterior. **c** Task 3

Figure 5 shows the tasks performed (Fig. 5a, b and c), as well as the direction of the axes used (Fig. 5b).

### Signal preprocessing

The signals were band-pass-filtered between 1 and 16 Hz, using a 5th order Butterworth [53]. The value of the signals was subtracted from its mean for the removal of linear trends. Following this, the resultant of the 3 axes of the accelerometer, gyroscope, and magnetometer sensors were calculated, using Eq. 1:

1 $$R=\sqrt{{s}_{x}^{2}+{s}_{y}^{2}+{s}_{z}^{2},}$$

where ${s}_{x}, {s}_{y}$ and ${s}_{z}$—measurements from the sensors along their respective axes; *R*—resultant.

### Description of parameters used

The parameters used were divided into the groups of amplitude, frequency, statistics and entropy and these are given in Eqs. 2 to 19 in Table 6. The features in the amplitude group are calculated in the time domain and are related to the values of the signal oscillations, which are related to how much the place, where the sensor was positioned, moved during the collection [54]. The parameters of the frequency group consist of showing which are the occurrences of an event during a given space in time calculated in the time domain, or determining frequency values related to energy measurements in the frequency domain [55, 56]. Entropy can reflect the disorder in a system, allowing for an understanding into the quantification of its complexity, when measuring the complexity of a system, values related to the uncertainty present within the sample window are possible [57]. Measures related to statistics aim to verify the form of data distribution and dispersion [56].

The equations are shown considering a signal *x*, composed of *N* samples, where each is represented by the index *i*: $x=\{{x}_{1}, {x}_{2}, {x}_{3}, \dots , {x}_{N}\}$.

### Classification between the young and elderly groups

In this study, the groups of young and elderly were determined in accordance with recommendations laid out by WHO, young participants were considered as aged below 60 years and elderly those above 60 years of age. Therefore, the young group was made up of the groups G1, G2, G3 and G4 and the elderly group by groups G5, G6, G7 and G8. The general features of the groups young and elderly are described in Table 5, where *N* represents the number of individuals from each group.

**Table 5:** General features of the groups young and elderly

Groups	Average, age ± sd (years)	*N*
Young	40.5 ± 12	53
Elderly	78.3 ± 10.5	46

The objective of this study was initially to investigate whether any feature from the signals was capable of differentiating between the young and elderly groups. Second, in cases where the feature makes the differentiation between the young and elderly groups, the test to confirm if it manages to verify gradual changes over aging is applied. The second step consisted of performing a pairwise test on the 8 groups used in this study, if the feature was able to differentiate the 8 groups from each other, the feature would be able to verify gradual changes during aging. The features were analyzed to confirm if they possessed normal distribution, through use of the Shapiro–Wilk test. As normal distribution was not proved for all features and groups, the Mann–Whitney test was applied, which considered as significant *p* < 0.05. If the features fail to detect gradual changes during aging (according to the age groups presented in groups 1 to 8 of this work), the features will be combined to obtain a single feature called the LDA-value, based on the LDA [26]. The correlation between the LDA-value and age will be investigated by means of the Pearson coefficient.

The features were calculated for the 3 executions of the tasks for each of the participants, in terms of the processing of the signals, the mean of each feature was calculated for each participant.

### Feature analysis

The 18 parameters presented on Table 6 were calculated for each of the 6 IMU sensors (3 sensors for each IMU) and for each task (3 tasks). Each calculation was considered as being a feature, totaling 324 features. Thus, the parameters, sensors and tasks form the set of features, that are not analyzed individually, to assess the differentiation between the groups of young and elderly individuals or in the creation of a linear model for aging.

**Table 6:** Parameters used for the extraction of features

Amplitude—(MAV, MAVFD, MAVSD, RMS, Peak)	
**MAV—mean absolute value** [38, 58, 59] $\mathrm{MAV}=\frac{1}{N}\sum_{i=1}^{N}\left|{x}_{i}\right|$ (2)	**MAVFD—mean absolute value of the first difference** [38, 39, 60] $\mathrm{MAVFD}=\frac{1}{N-1}\sum_{i=1}^{N-1}\left|{x}_{i+1}-{x}_{i}\right|$ (3)
**MAVSD—mean absolute value of the second difference** [38, 60] $\mathrm{MAVSD}=\frac{1}{N-2}\sum_{i=1}^{N-2}\left|{x}_{i+2}-{x}_{i}\right|$ (4)	**RMS—root mean square** [38, 58, 61, 62] $\mathrm{RMS}=\sqrt{\frac{1}{N}\sum_{i=1}^{N}{\left({x}_{i}\right)}^{2}}$ (5)
**Peak—maximum value of the vector, considering only positive values from the window** [38] $\mathrm{Peak}=\mathrm{max}{\left\{{x}_{i}\right\}}_{i=1}^{N}$ (6)	
**Frequency—(Zero Crossing, FMean, FPeak, F50, F80, Power3.5–7.5)**	
**Zero Crossing (ZC)** [38, 58, 59, 61] Given two consecutive samples *x*_*i*_ and *x*_*i*+*1*_, the counting of zero crossings, ZC, is increased if: $\left\{{x}_{i}>0 \mathrm{and} {x}_{i+1}<0\right\} or \left\{{x}_{i}<0 \mathrm{and} {x}_{i+1}>0\right\}$ (7)	**FMean—mean frequency** [38, 40, 41, 58, 62] $\mathrm{FMean}= \frac{\sum_{i=1}^{N}{(P}_{n}\left(i\right)*{f}_{n}\left(i\right)) }{\sum_{i=1}^{N}{P}_{n}\left(i\right)}$ (8) where ${P}_{n}$ is the power spectrum; ${f}_{n}$ is the vector frequency of ${P}_{n}.$
**FPeak—frequency at which maximum power occurs** [40, 41, 63] $FPeak=fn \mathrm{where} \left\{\mathrm{max}{\left\{{Pn}_{i}\right\}}_{i=1}^{N}\}\right.$ (9)	**F50—median frequency** [38, 40, 41, 58, 62, 63] $F50=\sum_{i=1}^{F50}{P}_{n}\left(i\right)= \sum_{F50}^{N}{P}_{n}\left(i\right)=\frac{1}{2}\sum_{i=1}^{N}Pn(i)$ (10)
**F80—total power frequency of Pn below 80%** [41, 64] $F80=frequency \mathrm{where} \left\{\sum_{i=1}^{F80}{P}_{n}\left(i\right)= 0.8*\sum_{i=1}^{N}Pn(i)\right\}$ (11)	**Power3.5–7.5—Power in frequency band 3.5–7.5 Hz** [42] $\mathrm{Power}3.5\_7.5=\sum_{{f}_{n}=3.5}^{{f}_{n}=7.5}Pn(i)$ (12)
**Statistic- (VAR, RANGE, INTQ, SKEW, KURTOSIS)**	
**VAR—variance** [38, 58, 61] $\mathrm{VAR}={\sigma }^{2}=\sum_{i=1}^{N}{\left({x}_{i}-\overline{x }\right)}^{2}$ (13) where $\overline{x }$—mean of the signal and $\sigma$—standard deviation	**RANGE—amplitude range** [26, 38] $\mathrm{RANGE}=\mathrm{max}{\left\{{x}_{i}\right\}}_{i=1}^{N}-\mathrm{min}{\left\{{x}_{i}\right\}}_{i=1}^{N}$ (14)
**INTQ—interquartile range** [38, 65, 66] $\mathrm{INTQ}=Q3-Q1$ (15) where ${Q}_{3}$ is the third quartile and ${Q}_{1}$ is the first quartile	**SKEWNESS—asymmetry** [39, 64, 67] $\mathrm{SKEWNESS}=\frac{\frac{1}{n}\sum_{i=1}^{N}{\left({x}_{i}-\overline{x }\right)}^{3}}{{\sigma }^{3}}$ (16)
**KURTOSIS—flattening** [39, 64, 67] $\mathrm{KURTOSIS}=\frac{\frac{1}{n}\sum_{i=1}^{N}{\left({x}_{i}-\overline{x }\right)}^{4}}{{\sigma }^{4}}$ (17)	
**Entropy—(approximate entropy and fuzzy entropy)**	
**ApEn—approximate entropy** [38, 61, 64, 68, 69] Entropy is an analysis tool used with goal of quantifying the regularity of a signal, returning a value between 0 and 2, where 0 indicates signal predictability based on previous values and 2 indicates signal unpredictability [70] Given a time series composed of *N* samples *{x(1), x(2), …, x(N)}* and *m* a sequence of vectors starting from *x(1)* until *x(N-m* + *1)*, defined by $x\left(i\right)=\left\{x\left(i\right), x\left(i+1\right), \dots , x\left(i+m-1\right)\right\}, i=1, \dots , N-m+1$ The distance between two vectors *x(i)* and *x(j)*, is defined as being the maximum distance between such elements—*d[x(i), x(j)].* For each value of *i* smaller than *N-m* + *1*, calculate ${C}_{i}^{m}$, defined as $number of j such as:(d\left[x\left(i\right), x(j)\le r)/(N-m+1)\right]$ Following this, calculate ${C}^{m}\left(r\right)$ given by ${C}^{m}\left(r\right)={\left(N-m+1\right)}^{-1}\sum_{i=1}^{N-m+1}ln{C}_{i}^{m}(r)$. The approximate entropy is given by (18) $ApEn\left(m,r,N\right)={C}^{m}\left(r\right)-{C}^{m+1}(r)$ (18) where *m*—window length; *r*—tolerance and *ln* is the natural logarithm	**FuzzyEn—fuzzy entropy** [38, 61, 71] Given a time series composed of *N* samples *{x(1), x(2), …, x(N)}* and *m* a sequence of vectors starting from *x(1)* until *x(N-m* + *1)*, calculate the degree of similarity between the vectors *x(i)* and *x(j)* defined by the fuzzy function ${d}_{[x\left(i\right),x\left(j\right)]}^{m}=\mu ({d}_{ij}^{m},r)$ where ${d}_{ij}^{m}$ is the largest difference between the elements of vectors *x(i)* and* x(j)*. For each vector *x(i)* calculate the mean of all degrees of similarity with its neighbors (*j ≠ i*) For each value of *i* smaller or equal to *N-m* + *1*, calculate ${P}_{i}^{m}$*(r)*, given by ${P}_{i}^{m}\left(r\right)={\left(N-m+1\right)}^{-1}\sum_{j=1}^{N-m}{d}_{[x\left(i\right),x\left(j\right)]}^{m}$ ${P}^{m}\left(r\right)={\left(N-m\right)}^{-1}\sum_{i=1}^{N-m}{P}_{i}^{m}\left(r\right)$ ${P}^{m+1}\left(r\right)={\left(N-m\right)}^{-1}\sum_{i=1}^{N-m}{P}_{i}^{m+1}\left(r\right)$ Fuzzy entropy is given by (19) $FuzzyEn\left(m,r,N\right)=\mathit{ln}{P}^{m}(r)-\mathit{ln}{P}^{m+1}(r)$ (19)

To enhance data reliability, the researchers employed outlier detection and removal techniques based on Eqs. 20 and 21 [72].

20 $$\mathrm{Lower}=Q1-1.5*\mathrm{INTQ},$$

21 $$\mathrm{Upper}=Q3+1.5*\mathrm{INTQ},$$

where INTQ—interquartile range (*Q3*–*Q*_1_); ${Q}_{3}$ is the third quartile; and ${Q}_{1}$ is the first quartile.

This critical step allowed these to identify and eliminate data points that significantly deviated from expected patterns, thus minimizing the influence of anomalies in the subsequent analysis.

The calculated features result in a large quantity of dimensions and may possess redundant information (high correlation); the redundant features do not significantly contribute to the calculation of the LDA-value. In order to reduce the dimensions and verify the redundancies of correlated features, feature reduction was performed using the Pearson correlation coefficient (r) making a pairwise comparison, removing, as such, redundant features that had high correlation, r values higher than 0.9 were considered as high correlation. This step reduced the number of features from 324 to 143 in the LDA-value calculation. To calculate the Pearson correlation, the “cor” function of the “R” software was used.

### Most important features selection

The quantity of features (a in Figs. 1 or 2), groups of features (b in Figs. 1 or 2), feature group proportion (c in Figs. 1 or 2), task (d in Figs. 1 or 2), sensors (e in Figs. 1 or 2), types of sensors (f in Figs. 1 or 2) and IMU (g in Figs. 1 or 2) that showed significant differences in the separation between young and old groups and the amount of relevant characteristics used for the calculation of the LDA-value were calculated and shown in Figs. 1 and 2, respectively. These results may indicate for future work on the features to be used, better positioning of IMUs and which sensors produce better results for classification of the algorithm used [23].

For the elaboration of Figs. 1 and 2a–g, we used Table 1 and 2, respectively, and the following strategy was adopted:

a—From Tables 1 or 2, the quantity of times a given feature was able to differentiate the groups of young and old was added.

b—From Tables 1 or 2, the quantity of times each group of parameters was able to differentiate the groups of young and old was added, considering each group of parameters as follows: amplitude (MAV, MAVFD, MAVSD, RMS, peak), frequency (zero crossing, FMean, FPeak, F50, F80, Power3.5–7.5), statistic (VAR, RANGE, INTQ, SKEW, KURTOSIS) and entropy (approximate entropy and fuzzy entropy).

c—All the statistical differences presented in Tables 1 or 2 were summed, then the amount of each parameter obtained for Figs. 1b or 2b is divided by this value. This data is important to show the proportional contribution of each parameter, since the amount of feature per parameter is not homogeneous.

d—The number of significant differences shown in Tables 1 or 2 between young and elderly people was summed for each Task (Task 1, Task 2 and Task 3), the values obtained were plotted.

e—All the times (Tables 1 or 2) in which the feature of each sensor (A1, A2, G1, G2, M1 and M2) showed statistical difference between young and elderly people were added up, the absolute value is shown in Figs. 1e or 2e.

f—The results of Figs. 1e or 2e were added for each type of sensor as follows: accelerometer (A1+A2), gyroscope (G1+G2), magnetometer (M1 + M2).

g—The results of Figs. 1e or 2e for each IMU were added as follows: IMU1 (A1 + G1+M1), IMU2 (A2+G2+M2).

### LDA-value analysis

Feature reduction eliminates redundant information, information with high correlation and therefore is repeated and eliminated. However, it is important to verify the relevance of the features, because even when eliminating redundancy, the number of features can reduce the performance of the classifiers [23]. Thus, the determination of the relevant features is carried out in two steps: in the first step, the redundant features are eliminated (using the Pearson correlation coefficient (r)) and in the second step, the remaining features are evaluated using the LDA and the Genetic Algorithm (GA) to verify the relevance. The LDA provides the rotation parameters of an imaginary axis (Eqs. 22, 23, 24, 25) for the GA that will control the positions of the imaginary axis in this space for a better selection of the position of the imaginary axis to discriminate the 8 groups.

LDA is a data classification and dimensional reduction method, which is able to project a multidimensional dataset onto one dimension, which represents the projection of all the features onto the imaginary axis. This projection results in a single value or a new feature, which in this study is called the LDA-value [73, 74]. In the present study, the LDA-value was used to verify if a calculated feature combination, can be used to discriminate the eight groups analyzed. The supplementary material shows the simplified algorithm “EstimateLDA_value”, for estimating the LDA-value.

### Main steps of the algorithm

- Data normalization—The input to the algorithm is a feature matrix (F), formed by concatenating the feature vectors (f) of each individual. There are 143 features that remain for each participant after the reduction process, each column of the matrix F is normalized between zero and one, an offset of 0.1 is added to the normalized vector to avoid division by zero during the calculation of the LDA-value. This step generates the matrix “$C$” of normalized data (normalized feature vectors (c) of each individual).
- Data representation (*calculate* (${R}_{0}, {\theta }_{0}$))—In this step, the data ($C$) are represented in the multidimensional angular coordinate space, in accordance with Eqs. 22, 23, 24, 25:
  22 $$p=\sqrt{{c}_{1}^{2}+{c}_{2}^{2}+{c}_{3}^{2}+\dots +{c}_{n}^{2}}$$
  23 $${R}_{0}=\left|p\right|$$
  24 $$\theta =\{{\theta }_{1}+{\theta }_{2}+{\theta }_{3}+\dots +{\theta }_{n-1}\}$$
  25 $${\theta }_{1}={\mathrm{tan}}^{-1}\left(\frac{{c}_{2}}{{c}_{1}}\right); {\theta }_{2}={\mathrm{tan}}^{-1}\left(\frac{{c}_{3}}{\sqrt{{c}_{1}^{2}+{c}_{2}^{2}}}\right); {\theta }_{n}={\mathrm{tan}}^{-1}\left(\frac{{c}_{n}}{\sqrt{{c}_{1}^{2}+{c}_{2}^{2}+\dots +{c}_{n-1}^{2}}}\right),$$

where *n*—number of features; *p*—radius; *R*_*0*_—module of *p*; and *Θ*—angle.

- Start of Genetic Algorithm (GA) implementation—At the start of the GA application, an initial population (${\widehat{\theta }}_{0}$) is defined, which is created from a sample of imaginary axes, for which the possible values vary from 0 to 2π. ${\widehat{\theta }}_{0}$ is used only in the first iteration, an updated population (${\widehat{\theta }}_{\mathrm{current}}$), will be used in the iterations that follow. The dimensional reduction consists of rotating an axis, created imaginarily, in the multidimensional space. The rotation of this imaginary axis opens the possibility of verifying the position for the projections of all the points (that is, all the individuals) on this axis, thus providing the best discrimination of the eight groups. The GA is used to find a better selection of the position of the imaginary axis. Therefore, the GA is used to find an optimized position for the imaginary axis, where the projection of each point on this axis produces a maximum separability between the groups in question. Consequently, Eq. 26 is used to carry out the projection of all existing individuals on each created imaginary axis, generating an A matrix. From the A matrix, it is possible to quantify ${E}_{z}$, which is the degree of discrimination of the groups along each imaginary axis through Eq. 27. The greater the value of ${E}_{z}$, the better the discrimination between groups on this axis. As the main objective of the GA is to find the position of the imaginary axis where ${E}_{z}$ is maximum, this will be the fitness function of the GA. The necessary calculations for using GA are defined below.
  o Projection of the data (*calculate* (LDA-*value*))—The data are projected onto a given axis, generating a scaler which is a new feature, this therefore is a linear combination of the old features called LDA-value. The multidimensional data are projected onto a unidimensional space, as depicted in Eq. 26, thus resulting in a matrix *A* composed of the values calculated in all iterations.
    26 $$LDA-value=p*\mathrm{cos}\left({\theta }_{1}+{\widehat{\theta }}_{1}\right)*\mathrm{cos}\left({\theta }_{2}+{\widehat{\theta }}_{2}\right)*  \cdots  *\mathrm\,{cos}\,({\theta }_{n-1}+ {\widehat{\theta }}_{n-1})$$

where $\hat{\theta }$—rotation angles which maximizes class separability.

- Accuracy estimator calculation (${E}_{z}Vec$ = *calculate* (${E}_{z}$ for each imaginary axis))—The projections onto *A* are used for an estimator of accuracy ${E}_{z}$, calculating as depicted in Eq. 27. The value of *E*_*Z*_ is calculated for each of the existing group pairs, the values obtained for each are added together, the value of the sum characterizes the separation of all the existing groups:
  27 $${E}_{z}=\sum_{i=1}^{\xi -1}\sum_{j=i+1}^{\xi }\left|\frac{({\overline{x} }_{i}-{\overline{x} }_{j})}{\sqrt{{\sigma }_{{x}_{i}}^{2}+{\sigma }_{{x}_{j}}^{2}}}\right|, z=1, 2, \dots , s,$$

where $\xi$—number of classes; ${\overline{x} }_{i}$ and ${\sigma }_{{x}_{i}}^{2}$ are the mean and variance of the *i*th class; ${\overline{x} }_{j}$ and ${\sigma }_{{x}_{j}}^{2}$ are the mean and variance of the *j*th class; and *S*—number of axes for the initial population.

The values of ${E}_{z}$ for each iteration are stored in a vector E_*z*_Vec, as shown in Eq. 28:

28 $${E}_{z}\mathrm{Vec}=\left[\begin{array}{c}{E}_{z=1}\\ {E}_{z=2}\\ .\\ .\\ .\\ {E}_{z=s}\end{array}\right]$$

o Selection by the roulette wheel technique (*K* = select (axes by the roulette wheel technique))—this selection creates the next generation, according to randomly selected individuals from the previous generation [75, 76]. This technique stochastically selects individuals from the population ${\widehat{\theta }}_{\mathrm{current}}$, using the probability that is proportional to the value *Ez*, originating in the matrix *K*.
o Generation of three descendants (${\widehat{\theta }}_{\mathrm{current}}$ = *Crossover_Mutation* (p_crossover_, p_mutation_))—In this step, three children are generated (${\widehat{\theta }}_{\mathrm{child}1}, {\widehat{\theta }}_{\mathrm{child}2} \,and\, {\widehat{\theta }}_{\mathrm{child}3})$ from two parents of the actual generation (${\widehat{\theta }}_{\mathrm{parent}1} and\, {\widehat{\theta }}_{\mathrm{parent}2})$. The children are obtained from the application of the crossover and mutation on matrix *K,* with the crossover and mutation probabilities passed on as parameters to the GA, *p*_crossover_ and *p*_mutation_, respectively. Only the two best children, in accordance with ${E}_{z},$ are selected. Equations 29, 30 and 31:
  29 $${\widehat{\theta }}_{\mathrm{child}1}=1.5{\widehat{\theta }}_{\mathrm{parent}1}-0.5{\widehat{\theta }}_{\mathrm{parent}2}$$
  30 $${\widehat{\theta }}_{\mathrm{child}2}=0.5{\widehat{\theta }}_{\mathrm{parent}1}+0.5{\widehat{\theta }}_{\mathrm{parent}2}$$
  31 $${\widehat{\theta }}_{\mathrm{child}3}=-0.5{\widehat{\theta }}_{\mathrm{parent}1}+1.5{\widehat{\theta }}_{\mathrm{parent}2}.$$
  o Random change—According to mutation probability, the angle of rotation of a given individual on matrix *K* is randomly modified, giving origin to a new population (${\widehat{\theta }}_{\mathrm{current}})$.
  • End of GA implementation (*if* g ≤ epochs)—When the number of predetermined epochs (passed on as parameters to the GA) is reached, the imaginary axis that has the highest value of ${E}_{z}$ is selected, thus finalizing the GA step.
  • Selection of relevant features (${E}_{z}\mathrm{Vec}$ = *estimate_relevant_features* ($C$))—from the selected imaginary axis, the relevance of each feature is verified, the features considered irrelevant (relevance less than 1% of the accuracy estimator ${E}_{z}$) are eliminated (${C}_{\mathrm{new}}$ = *eliminate irrelevant features* ($C$)) and a new $R$ and $\theta$ is calculated from these features.
  • Estimate of the LDA-value (*calculate* (*LDA-value*))—the relative points of the relevant features are projected onto the imaginary axis and the LDA-value is calculated (Eq. 25).

Table 7 presents the input values for the GA utilized for the estimation of the LDA-value.

**Table 7:** Parameters used for estimating the LDA-value

Parameters	Values	Description
$\mathrm{epochs}$	50,000	Number of epochs
$s$	50	Number of axes in the initial population
$\xi$	8	Number of groups
*p*_mutation_	0.1	Probability of mutation
*P*_crossover_	0.8	Probability of crossover

### Supplementary Information

**Additional file 1: Figure S1.** Simplified algorithm for estimating the LDA-*value*.

## Data Availability

The datasets generated in the current study are not publicly available due to the ethical restrictions preventing public sharing of data. A non-identified set may be requested after approval from the Review Board of the Institution. Requests for the data may be sent to the corresponding author.

## Abbreviations

LDA: Linear discriminant analysis
WHO: World Health Organization
ML: Machine Learning
CONEP: National Commission of Ethics in Research
TREMSEN: Precise Tremor Sensing Technology
INPI: National Institute of Industrial Property
NIATS: Center for Innovation and Technological Assessment in Health
UFU: Federal University of Uberlandia
IMU: Inertial measurement unit
A1: Accelerometer of IMU1
G1: Gyroscope 1 of IMU1
M1: Magnetometer 1 of IMU1
A2: Accelerometer 2 of IMU2
G2: Gyroscope 2 of IMU2
M2: Magnetometer 2 of IMU2
MAV: Mean absolute value
MAVFD: Mean absolute value of the first difference
MAVSD: Mean absolute value of the second difference
RMS: Root mean square
ZC: Zero crossing
FMean: Mean frequency
FPeak: Frequency at which maximum power occurs
F50: Median frequency
F80: Total power frequency of Pn below 80%
Power3.5–7.5: Power in frequency band 3.5–7.5 Hz
VAR: Variance
INTQ: Interquartile range
ApEn: Approximate entropy
FuzzyEn: Fuzzy entropy
GA: Genetic Algorithm
EMG: Electromyography
EEG: Electroencephalography
LTS: Lie-to-stand
CNPq: National Council for Scientific and Technological Development
CAPES: Coordination for the Improvement of Higher Education Personnel
FAPEMIG: Foundation for Research Support of the State of Minas Gerais
