# Supplementary figures and images for: A hybrid linear discriminant analysis and genetic algorithm to create a linear model of aging when performing motor tasks through inertial sensors positioned on the hand and forearm
Source: Biomed Eng Online. 2023 Oct 16;22:98. doi: 10.1186/s12938-023-01161-4 (PMC10580547; doi:10.1186/s12938-023-01161-4)

**
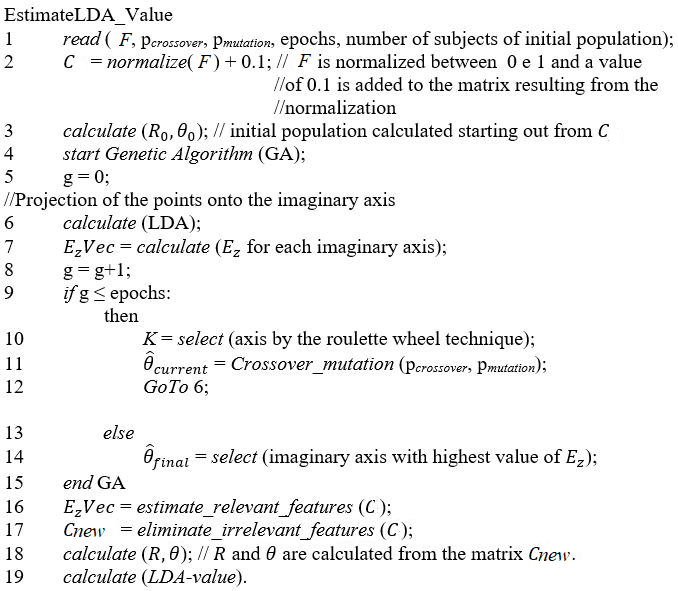
**

**Figure S1** – Simplified algorithm for estimating the LDA-*value*.

Supplement: Supplementary file 1 — Additional file 1: Figure S1. Simplified algorithm for estimating the LDA-value. [file 12938_2023_1161_MOESM1_ESM.docx]
